# Dr, Wm. Roberts Has Died of His Wounds Received at the Battle of Molino Del Rey

**Published:** 1848-01

**Authors:** 


"Dr, Wm. Roberts has died of his wounds received at the battle of Molino del Rey."

Dr Roberts was, we think, a native of Montreal, and educated originally for the church; but in 1837 he commenced the study of medicine at Auburn, Cayuga Co., in the office of Dr. Hamilton, and in 1840 graduated at the University of Pennsylvania, Philadelphia. Soon after, he received an appointment as assistant surgeon in the army, and served several months in the Florida compaign, in Gen. Read's Brigade. Upon the disbanding of this corps, he settled as a practioner of medicine and surgery at Brunswick in Georgia. In August, 1843, he was again admitted into the regular army as an assistant surgeon, where he remained until his death.

Dr. Roberts was a man of superior talents, and although not more than thirty years of age, his attainments were varied and extraordinary. In addition to a superior English education, he added a complete knowledge of the French; with also a sufficient acquaintance with the Latin and Greek to enable him to read the most difficult authors with facility. And while pursuing his medical studies he supported himself chiefly by teaching small private classes in these several branches. As a student of medicine, he had scarcely a rival, having by untiring industry, aided by uncommon greatness of apprehension and strength of memory, mastered more of the details of the several branches of our profession than any man of his age we have known. His private virtues were also of the most excellent character—he was humane and kind to a proverb, and to all his circle of friends he endeared himself most by his tender attachment and care for a widowed mother and an only sister.

Dr. Roberts was attached to the "veteran 5th regiment" of Worth's division, and was on the field in nearly all the fearful battles in which this regiment was engaged. It was always his desire to distinguish himself as a soldier, as well as in his capacity as a medical officer, and we presume it was at his own request that we find him leading a company in the charge upon Cherubusco on the 20th of August, for his courage and valuable services on which occasion he received a most flattering notice in the official despatches of his commanding officer. He received, however, his fatal wound at the assault upon Molino del Rey several days later. Of the character of his wounds, and the particulars of his death, we have no information, but we have no doubt he died as he had always lived, in trustful reliance upon his Heavenly Father.

